# Pressure for change: can we continue to ignore the lack of evidence for blood pressure augmentation to treat delayed neurological deficit following subarachnoid haemorrhage?

**DOI:** 10.1186/s13613-024-01273-7

**Published:** 2024-04-01

**Authors:** Ronan O’Leary, Jonathan P. Coles, Lara Prisco

**Affiliations:** 1 Neurosciences and Trauma Critical Care Unit, Cambridge University Hospitals, Cambridge, UK; 2 Perioperative, Acute, Critical Care, and Emergency Medicine Section, Department of Medicine, University of Cambridge, Cambridge, UK; 3 Nuffield Department of Clinical Neurosciences, University of Oxford and the Neurocritical Care Unit, Oxford University Hospitals, John Radcliffe Hospital, Oxford, UK

Recent guidelines [1, 2] starkly emphasise the sparce evidence in support of augmented hypertension (AH) to treat delayed cerebral ischaemia (DCI) following aneurysmal subarachnoid haemorrhage (aSAH), while other studies highlight the potential risks from adverse events [3, 4].

Macrovascular vasospasm (MV), narrowing of the large cerebral arteries, occurs in 30–70% of patients following aSAH and is thought to cause cerebral infarction. Supraphysiological augmented hypertension (AH) is widely used in the belief that it will overcome the impairment in cerebral blood flow thus restoring perfusion. However, concerns about efficacy and harm arise from studies questioning the relationship between MV and ischaemia-infarction, as well as evidence of iatrogenic harm from haemodynamic manipulation. Furthermore, as our knowledge of cerebrovascular physiology has developed, it seems implausible that this approach encapsulates all configurations of cerebrovascular perfusion.

A secondary analysis of the CONSCIOUS-1 study, which examined the natural history of cerebral ischemia following aSAH, illuminates this uncertainly and should prompt reflection about the current approach to this disease [5]. This study did not find a causative relationship between MV, DCI, and infarction, concluding that infarction arises from vasospasm-dependent and -independent mechanisms. This work highlights the complex derangements responsible for ischaemia-infarction and, crucially, confirms that DCI occurs both in the presence and absence of MV.

In contrast, much of the evidence in support of AH is from case reports and small cohort studies where neurological improvements have been associated with increases in blood pressure. This approach, whilst plausible in certain scenarios, is fraught with methodological bias; patients with aSAH have fluctuating neurology, sedation resolves unpredictably, and optimisation of routine physiological parameters may be enough to improve neurology. It is precisely these types of diagnostic-therapeutic challenges which benefit from well-designed, prospective clinical studies but these are almost wholly absent from the DCI literature [6].

As such, three knowledge gaps must be addressed via prospective clinical research to recommend AH as an effective and safe management strategy to treat DCI.

## Gap 1: standardised diagnostic criteria

Treatment for MV may necessitate AH but clinicians frequently encounter diagnostic uncertainty when patients present with suspected DCI. This lack of precision confounds the construction of a patient-centric analysis to determine how to use AH safely. Moreover, where a ‘MV first’ approach is used, it neglects other causes of neurological deterioration including seizures, sepsis, spreading depolarization, hypovolemia, disequilibrium syndromes, microvascular thrombosis, and delirium.

Once suspected, the tools used to diagnose DCI/MV, and to assess response to AH, lack standardisation. This disproportionally impacts the treatment of patients at the more severe end of the disease spectrum who are intubated and therefore unable to be assessed clinically. Altered computed tomography perfusion (CTP) scan temporal parameters are frequently felt to be suggestive of ischaemia and thus prompt the setting of arbitrary blood pressure targets. However, the baseline ‘normal’ CTP in any patient with DCI is frequently not known and it is not surprising that the combination of a chronically diseased cerebrovascular system, the interventions to secure the aneurysm, cerebral oedema, and MV interact to alter blood flow around the brain leading to the frequently observed alternations in temporal parameters, but this does not necessarily mean that AH will provide benefit. This ad hoc approach contrasts with the use of CTP in other diseases, such as ischaemic stroke, where clear diagnostic criteria define the extent of the ischaemic penumbra to allow safe, efficacious titration of mechanical thrombectomy.

In contrast to aSAH, radiological presence of MV does not have widespread utility as a threshold for the use of AH. For example, AH is not used routinely where MV is observed in posterior reversible encephalopathy syndrome (PRES) where it is postulated that supraphysiological blood pressure can trigger MV [7], or for cerebral angiopathy and vasoconstriction seen in pre-eclampsia.

## Gap 2: evidence demonstrating therapeutic benefit

Clazosentan, an endothelin receptor antagonist, decreased vasospasm in the CONSCIOUS − 1, -2, and − 3 studies but did not reduce the incidence of cerebral infarction, nor improve outcome [5]. Similar findings have been shown for angioplasty and, fascinatingly, while the arterial vasodilator nimodipine improves outcome following aSAH it does not reduce MV. These data suggest that MV may only play a partial role in the development of DCI and infarction and that any beneficial effect of AH may not come from overcoming the effects of MV.

More worryingly, AH itself could be harmful to uninjured regions of the brain. The proximal pressure achieved by AH may result in heterogenous patterns of perfusion due to the interaction between vasoactive medications, augmented blood pressure, and variably impaired cerebrovascular autoregulation. This conceivably results in ischaemia via exacerbation of physiological protective myogenic vasoconstriction, diversion of blood flow to non-spastic segments leading to hyperaemia and vasogenic oedema, or no change in flow - and therefore no benefit - due to intact cerebral autoregulation [8]. Any effective DCI treatment must reverse a potential deficit in flow and restore perfusion to ischaemic parts of the brain without creating any new perfusion deficits. It is not yet clear whether AH meets this crucial objective.

A more speculative anxiety is that adrenoreceptor agonists may act on adrenoreceptors in the cerebrovascular system to cause vasoconstriction [9–11]. Adrenoreceptors are abundantly expressed throughout the brain, particularly in the posterior circulation and it is uncertain what effect exogenous catecholamines have, especially in the presence of a disrupted blood-brain barrier. Small perturbations of blood flow to these regions in an already vulnerable brain could have substantial impact on outcome.

## Gap 3: evidence that the harms associated with AH are justified

Across a variety of clinical scenarios where supraphysiological doses of vasoactive medications are used there has been a consistent association with harm. It has yet to be established how much extra-cranial harm accumulates when AH is used to treat DCI/MV and whether this is mitigated by any beneficial effect derived from improved perfusion past MV. These foci of harm include catecholamine mediated injury to the heart, pulmonary circulation, and other organs. These effects may exacerbate the overall burden of injury caused by critical illness, further impair oxygen delivery to the brain, and worsen patient-centred outcomes.

There is also scant understanding of the relationship between AH and the common, simultaneously occurring, extra-cranial complications associated with aSAH; such as neurogenic cardiomyopathy and pulmonary oedema, hyponatraemia, and venous thromboembolism.

## Concluding comments

Until the gaps in knowledge articulated here have been addressed, AH to treat suspected DCI following aSAH should only be used within a structured, stepwise framework titrated to an objective, clinically important response. This must be sensitive to the significant potential complications whilst ensuring that other causes of neurological deterioration are not neglected.

These gaps will require internationally coordinated collaboration, research, and guideline setting. Research should focus, in the first instance, on establishing a clear diagnostic substrate for DCI to allow case selection on which prospective clinical studies can be designed and constructed. Outcomes should be defined using long-term measures of cognitive function which are patient-centric rather than surrogates of cerebral blood flow in the first days after ictus.

## Data Availability

Not applicable.
